# Ileal perforation due to *Ascaris lumbricoides*: an incidental finding in ileostomy complicated by peritonitis

**DOI:** 10.1093/jscr/rjaf647

**Published:** 2025-09-02

**Authors:** Jesus Sebastian Luna Medrano, Maylin Abigail Matienzo Rafael, Carlos Rojas-Arana, Astrid Geraldine Carrión Cuellar, Moises Miguel Florentino Mota, Paolo Augusto Romero Merino, Erick Villacis

**Affiliations:** Universidad Nacional José Faustino Sanchez Carrión, Av. Mercedes Indacochea 609, Huacho 15136, Peru; Universidad Cesar Vallejo, Av. Alfredo Mendiola, 6232, Los Olivos, Peru; Universidad Cientifica del Sur, Antigua Panamericana Sur 19, Villa EL Salvador 15067, Peru; Universidad Autonoma de Barcelona, Plaça Cívica, 08193 Bellaterra, Spain; Universidad Autonoma de Santo Domingo, Ciudad Universitaria. Avenue Alma Mater 1355, Santo Domingo 10014, Dominican Republic; Hospital Clinico Universitario de Valladolid, Av. Ramón y Cajal, 3, 47003 Valladolid, Spain; Universidad Catolica de Santiago de Guayaquil, R39W+98W, Av. Pdte. Carlos Julio Arosemena Tola, Guayaquil 090615, Ecuador

**Keywords:** ascariasis, peritonitis, intestinal perforation, helminthiasis, ileostomy

## Abstract

Ascariasis, caused by *Ascaris lumbricoides*, is the most common helminthic infection worldwide, mainly in developing countries. Although intestinal obstruction is its most frequent complication, intestinal perforation with peritonitis is rare and life-threatening. A 45-year-old woman from a rural area presented with cramping abdominal pain, intermittent fever, and asthenia for 3 months. Emergency laparotomy revealed generalized peritonitis due to ileal perforation, managed with ileal resection and ileostomy. Postoperative complications included respiratory failure, septic shock, and three reoperations for eventration, adhesive syndrome, and hemorrhage. On postoperative Day 33, a 20 cm *A. lumbricoides* worm was expelled through the ileostomy. Intestinal perforation likely resulted from pressure-induced ischemia by a worm mass. Diagnosis can be delayed due to nonspecific symptoms and absent eosinophilia. Ascariasis should be considered in the differential diagnosis of intestinal perforation in endemic areas. Early surgical intervention, clinical suspicion, and preventive measures are key to improving outcomes.

## Introduction

Ascariasis, caused by the nematode *Ascaris lumbricoides*, affects ~1.4 billion people in developing countries and is the most common helminthic infection worldwide, with an estimated 20 000 deaths annually [1]. Adult worms typically reside in the jejunum. Infection occurs via ingestion of embryonated eggs, which hatch in the small intestine; larvae migrate through the lungs before maturing in the gastrointestinal tract [2]. While mechanical intestinal obstruction is the most frequent complication, intestinal perforation is a rare cause of acute abdomen, representing ~5.3% of adult intestinal obstructions [3]. We report a rare case of ileal perforation and peritonitis due to *A. lumbricoides*, identified incidentally during postoperative evaluation.

## Case description

A 45-year-old woman from a rural area, with no relevant medical history, presented with a three-month history of cramping abdominal pain in both iliac fossae, accompanied by intermittent fever and asthenia, unrelieved by analgesics.

Physical examination revealed abdominal distension, diffuse tenderness, and a positive Blumberg’s sign. Laboratory tests showed a white blood cell count within normal limits (5340/mm^3^) with a predominance of segmented neutrophils (4600/μl).

**Figure 1:**
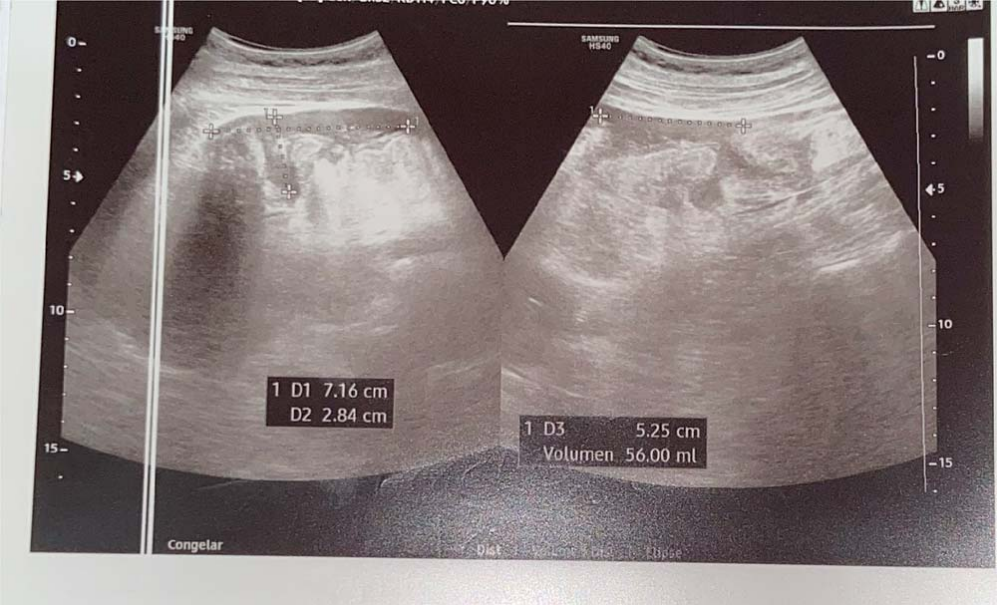
Ultrasound revealing revealed a small amount of free fluid in the perihepatic, perisplenic, and right flank interloop regions.

An abdominal ultrasound was performed, as it was the only imaging modality available at the local hospital. The study revealed dilated small bowel loops with a maximum diameter of 7 cm and bowel wall thickness of 2.8 cm, findings consistent with mechanical intestinal obstruction. Free intraperitoneal fluid was estimated at 56 ml. Echogenic tubular structures with parallel lines were observed within the intestinal lumen, and there was focal irregular thickening of the distal ileal wall with punctate hyperechoic foci and reverberation artifacts,suggestive of ileal perforation (Fig. 1). A CT scan was not performed due to limited resources, prompting emergency exploratory laparotomy based on clinical and laboratory findings.

Intraoperatively, 800 ml of purulent fluid was found in the peritoneal cavity, along with edematous intestinal loops and a 1 cm perforation in the ileum ~15 cm from the ileocecal valve. The final diagnosis was generalized peritonitis. Surgical management included an ileal resection with terminal ileostomy, appendectomy, peritoneal lavage, and placement of a laminar drain.

In the immediate postoperative period, the patient developed acute respiratory failure requiring mechanical ventilation, abdominal septic shock, capillary leak syndrome with hypoalbuminemia (1.68 g/dl), and hyponatremia (128 mEq/L). She was transferred to the intensive care unit for management.

The patient remained stable until postoperative Day 8, when she developed bilious vomiting, followed by a grade 3 eventration 24 hours later. Surgical repair revealed an 8 cm aponeurotic dehiscence, 100 ml of inflammatory fluid, an intestinal loop protruding through the midline wound, fibrin deposits, and parieto-enteric adhesions. Adhesiolysis, peritoneal lavage, and abdominal wall closure were performed.

Five days later, she developed vulvar edema, managed conservatively following gynecologic consultation. Two weeks post-eventration repair, she underwent reoperation for suspected residual abscess and adhesive syndrome due to prolonged ileus. Intraoperative findings included 500 ml of serous fluid in the right upper quadrant, hepatoperitoneal adhesions, 70% intestinal rotation, and a coarse hepatic surface. Adhesiolysis, intestinal devolvulation, and lavage were performed.

**Figure 2:**
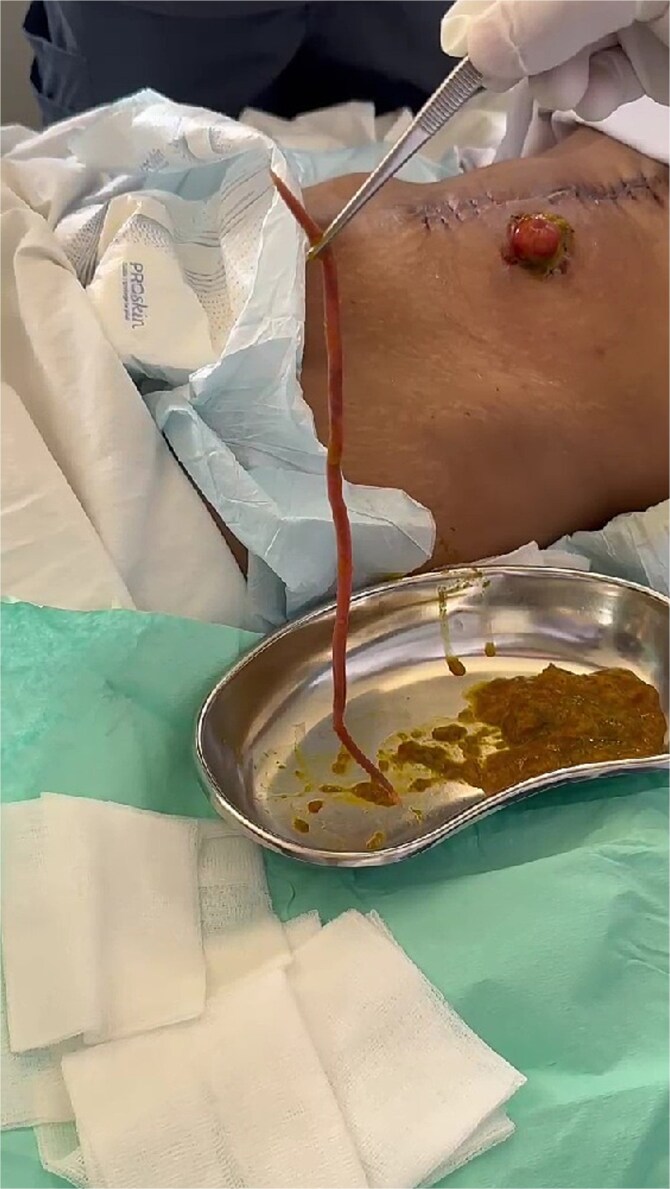
*Ascaris lumbricoides* extracted from ileostomy bag.

Postoperatively, serohematic wound discharge persisted. Two days later, active epigastric bleeding necessitated a fourth surgery, revealing 120 mL\l of clotted blood adherent to the rectus aponeurosis; hemostasis was achieved. On postoperative day 33, the patient spontaneously expelled a 20 cm *A. lumbricoides* specimen via the ileostomy. Albendazole 400 mg was administered as a single dose, with no prior empirical anthelmintic therapy. The patient subsequently improved and was discharged with outpatient follow-up arranged (Fig. 2 and Video S1).

## Discussion

*A. lumbricoides* is one of the most prevalent intestinal parasites worldwide, mainly affecting tropical and subtropical regions with poor sanitation [4]. It affects >700 million people, particularly children and rural populations in low-income settings [5]. Transmission occurs primarily through ingestion of embryonated eggs in contaminated food or water [6].

Most cases are asymptomatic or present with nonspecific symptoms such as abdominal pain, bloating, nausea, or diarrhea [7]. Severe cases may lead to complications including intestinal obstruction, appendicitis, pancreatitis, cholecystitis, or intestinal perforation [8].

Our patient’s prolonged colicky pain and asthenia could reflect nutrient competition by the parasite and altered microbiota [9, 10]. The absence of eosinophilia, typically seen during larval migration [11], contributed to delayed suspicion of parasitic infection.

Intestinal obstruction is the most common complication of ascariasis, mainly affecting the distal ileum due to its narrow lumen and the anatomical barrier of the ileocecal valve [12]. In contrast, intestinal perforation is rare but serious, representing up to 10% of surgically treated cases [13, 14].

The mechanism of intestinal perforation in ascariasis remains a subject of debate: Perforation can result from continuous pressure necrosis by a worm mass or preexisting ulcer that allow larval invasion [2, 3]. Current evidence supports pressure necrosis as the primary cause rather than active larval invasion. Formation of a large *Ascaris bolus* leads to luminal obstruction, bowel distension, and progressive compromise of the intestinal wall vascular supply. Prolonged ischemia results in focal necrosis and eventual perforation, often occurring in previously healthy bowel segments [15].

In contrast, the theory of larval invasion has limited support, as *A. lumbricoides* lacks anatomical structures to penetrate intact mucosa actively. Perforations attributed to this mechanism are more likely secondary to pre-existing ulcerations or inflammatory lesions through which the parasite escapes rather than invades [16].

While eosinophilia is a characteristic feature of helminth infections, its absence does not preclude chronic *A. lumbricoides* infestation. Eosinophil elevation is typically observed during larval migration due to IL-4 and IL-5 activity, but levels often normalize in chronic phases as a result of parasite-driven immunomodulation. This attenuated immune response facilitates parasite survival and reduces clinical manifestations. Therefore, in endemic settings, diagnosis should not rely solely on eosinophil counts but should incorporate imaging, stool examination, or antigen detection [17, 18].

Limitations of this report include lack of long-term follow-up, lack of stool exams or serology preoperatively and absence of histopathological examination of the resected bowel.

## Conclusion

This case underscores *A. lumbricoides* as an uncommon but important cause of intestinal perforation and peritonitis. It highlights the need to include parasitic infections in the differential diagnosis of acute abdomen, even in the absence of eosinophilia. Prompt surgical management, appropriate antiparasitic therapy, and strengthened public health measures are essential, particularly in endemic regions where such infections are often underdiagnosed.

Implementation of community-based deworming programs is strongly recommended as a public strategy to prevent *A. lumbricoides* infection and its complications in endemic areas following the World Health Organization guidelines on the control of soil-transmitted helminthiasis, which emphasize periodic mass drug administration as an effective preventive measure.

## Supplementary Material

Video_Ascaris_rjaf647
